# Revisiting awake prone positioning in COVID-19

**DOI:** 10.1186/s13613-025-01539-8

**Published:** 2025-08-06

**Authors:** Yisheng Cao, Qianqian Mao

**Affiliations:** 1 Department of Anorectal Surgery, Ningbo No.2 Hospital, Ningbo, Zhejiang China; 2 Department of General Surgery, Cixi Maternity and Child Health Care Hospital, No. 1288, Erzaotang Road, Bashaluo Street, Cixi City, Ningbo, Zhejiang China

## To the editor,

We carefully read the recent secondary analysis by Sun et al. [1] published in Annals of Intensive Care. The authors used data from a multicenter randomized controlled trial to investigate the relationship between daily duration of awake prone positioning (APP) and the risk of intubation or death within 28 days (APP failure) among patients with COVID-19-related acute hypoxemic respiratory failure. They concluded that maintaining APP for 8–12 h per day may be optimal. However, we believe that addressing several key factors would enhance the scientific depth and practical relevance of the study’s conclusions.

First, the study included 408 patients but did not report the COVID-19 wave or SARS-CoV-2 variant types, nor did it provide information on vaccination status or timing. Numerous studies have shown that different variants have significant differences in pathogenicity, extent of lung involvement, and mechanisms of hypoxemia [2]. For instance, the Omicron variant is associated with a lower rate of ARDS and reduced need for respiratory support compared to the Delta variant [3]. In addition, vaccination markedly reduces the risk of severe illness and death in COVID-19 patients [4, 5]. The absence of these key contextual variables may introduce considerable confounding and limit the ability to attribute observed effects solely to APP duration.

Second, the study noted that 75.6% of patients in the APP success group received conventional low-flow oxygen therapy, compared to only 31.4% in the failure group. In contrast, high-flow nasal cannula (HFNC) and non-invasive ventilation (NIV) were more frequently used in the APP failure group (46.7% and 21.9% respectively). Multivariate analysis also identified “non-invasive respiratory support” as an independent risk factor (HR = 6.04, 95% CI: 1.68–21.78). However, the authors did not further explore whether there was an interaction between APP duration and type of respiratory support. Previous studies using tools like electrical impedance tomography have shown that different modes of respiratory support can result in distinct patterns of lung ventilation and recruitment during posture changes [6]. Future research incorporating respiratory support modality into subgroup analyses or interaction models with APP duration could help identify patient groups that may benefit most from specific strategies, thereby guiding tailored interventions.

Finally, while the study found that maintaining APP for 8–12 h per day was associated with the best prognosis, such prolonged prone positioning poses practical challenges in real-world clinical settings. Elderly, frail patients or those with comorbid conditions such as chronic pain or anxiety may struggle to maintain this position for extended periods, affecting adherence and treatment completion [7]. To improve the real-world applicability of APP, future studies could explore diversified postural strategies (lateral positioning, intermittent APP), phased intervention protocols, and the integration of supportive measures such as sedation, pain management, psychological support, and involvement of family or social workers. These multidimensional approaches could not only improve patient comfort and experience but also broaden the scope of APP applicability to a wider patient population, enhancing feasibility and clinical implementation without compromising efficacy.

In conclusion, we commend Sun et al. for their valuable work and look forward to future prospective studies with more refined stratified designs to advance the precision use of APP across diverse patient populations.

## Data Availability

My manuscript has no associated data.
